# *APOE* Gene Polymorphism and Risk of Coronary Stenosis in Pakistani Population

**DOI:** 10.1155/2015/587465

**Published:** 2015-03-26

**Authors:** Asma Naseer Cheema, Attya Bhatti, Xingbin Wang, Jabar Ali, Mikhil N. Bamne, F. Yesim Demirci, M. Ilyas Kamboh

**Affiliations:** ^1^Atta-Ur-Rahman School of Applied Biosciences, National University of Sciences & Technology, H/12, Islamabad 44000, Pakistan; ^2^Department of Human Genetics, University of Pittsburgh, Pittsburgh, PA 15261, USA; ^3^Department of Cardiology, Lady Reading Hospital, Peshawar 25000, Pakistan

## Abstract

Genetic variation in lipid regulatory genes, particularly *APOE*, significantly influences the risk of coronary artery disease (CAD). This study aimed to assess the association between *APOE* polymorphism and angiographically assessed coronary stenosis in Pakistani population. A total of 695 subjects (22.3% female, mean age = 54 ± 11 years) presenting with chest pain were enrolled after obtaining written informed consent. CAD stenosis/extent was assessed by angiography. Patients were classified as having severe stenosis (≥70%), moderate stenosis (30–69%), and mild stenosis (<30%). CAD patients with ≥70% stenosis (*n* = 491) were further categorized based on possessing one, two, or three vessel diseases to assess the disease extent. Genomic DNA from leukocytes was isolated with DNA purification kit (Qiagen) and *APOE* polymorphisms (E2/E3/E4) were determined using TaqMan assays. Six hundred and seventy-two of 695 subjects were successfully genotyped. The frequency of *APOE∗4* carriers (3/4 and 4/4 genotypes) was significantly higher in severe stenosis group (≥70%) as compared to mild group (<30%) (22.8% versus 13.01%; *P* = 0.01). In multiple regression, the odds ratio for *APOE∗4* carriers to develop ≥70% stenosis was 2.16 (95% CI: 1.29–3.79; *P* < 0.005). In conclusion, the presence of *APOE∗4* allele is a significant risk factor to develop severe coronary stenosis (>70%) among Pakistanis.

## 1. Introduction

Coronary artery disease (CAD) is a complex and multifactorial disorder involving the interplay of both genetic and environmental factors. Despite significant improvement in clinical management of the CAD, it is still reported as the most common cause of adult deaths worldwide [1]. Geographically, there is asymmetrical pattern of CAD distribution such that the disease burden is projected to be double by 2020 in developing countries as compared to the only 50% rise in developed countries [2]. The integrative approach of analyzing the disease both clinically and genetically provides the opportunity to disentangle the complex interactions underlying disease pathways. While lifestyle modification has reduced the mortality rate, the candidate gene approach has provided new insights for exploring diagnostic and therapeutic approaches. The important role of lipid regulatory genes in CAD pathogenesis has been an essential aspect of research for the past many years [3, 4]. Apolipoprotein E (ApoE protein;* APOE* gene), one of the key regulators of lipid metabolism, plays an important role in the uptake of ApoE-containing lipoprotein particles by the cells. There is a common ApoE protein polymorphism with three alleles,* APOE*∗*2*,* APOE*∗*3*, and* APOE*∗*4*, which are differentiated by amino acid substitution at codons 112 and 158 in exon 4. ApoE2 has cysteine at both positions; E3 has cysteine at 112 and arginine at 158 while E4 has arginine at both positions [5]. The three alleles show marked variation in distribution among different racial groups and are associated with variation in plasma cholesterol levels in the general population [6].* APOE* polymorphism is also associated with atherosclerosis where the* APOE*∗*4* allele is linked with elevated risk of CAD [7, 8]. However, epidemiological studies have shown inconsistent association of* APOE* polymorphism with clinical CAD [9–12].

The aim of our study was to seek the association between* APOE* polymorphism and angiographically assessed coronary stenosis among Pakistanis, which to the best of our knowledge has not been reported previously.

## 2. Materials and Methods

### 2.1. Subjects

A total of 695 subjects were enrolled at the Lady Reading Hospital, Peshawar, after obtaining written and oral consent. The institutional review board and ethical committee of National University of Sciences & Technology approved the study. The study was designed to enroll patients presenting with chest pain or suspected to have clinical myocardial ischemia. The study participants were classified as having severe stenosis (≥70%), moderate stenosis (30–69%), and mild stenosis (<30%). CAD patients with ≥70% stenosis were further categorized based on possessing one, two, or three vessel diseases to assess the disease extent. The participants with cardiomyopathy, NYHA class IV CCF, valvular or congenital heart disease, and recent coronary angioplasty or coronary bypass surgeries were excluded from the study.

### 2.2. *APOE* Genotyping

Genomic DNA was isolated from leukocytes using DNA purification kit (Qiagen, Valencia, CA). Genotypes for two* APOE* single-nucleotide polymorphisms (SNPs), rs429358 (*E*4) and rs7412 (*E*2), were determined by TaqMan SNP genotyping assays on ABI Prism 7900HT Sequence Detection System (Life Technologies, Grand Island, NY). To estimate the assay error rate, 10% of samples were randomly selected and included as duplicates in genotyping run. There was only one discrepant sample, which was removed from the final analysis. The genotype outputs from the two SNPs were converted into the conventional six* APOE* genotypes (22, 23, 33, 34, 24, and 44) based on three alleles (*APOE*∗*2*,* APOE*∗*3*, and* APOE*∗*4*).

### 2.3. Statistical Analysis

Differences in mean values for age, body mass index (BMI), and lipid levels were analyzed using one-way ANOVA. Chi-square (*χ* ^2^) test was performed to assess the distribution of sex, smoking, and family history of CAD among the three-stenosis groups.* APOE* allele frequencies were calculated by allele counting. The Hardy-Weinberg equilibrium was tested by a *χ* ^2^ goodness-of-fit test. For association analysis between* APOE* and CAD severity,* APOE* genotypes were collapsed into three groups: E2 (2/2 and 2/3 genotypes), E3 (3/3 genotype), and E4 (3/4 and 4/4 genotypes). Because of the known opposite effects of the* APOE*∗*2* and* APOE*∗*4* alleles on the risk of CAD, we omitted individuals with the 2/4 genotype from the analysis. The Cochran-Armitage (C-A) trend test was applied in the analysis of the association between* APOE* and CAD severity [13]. Multiple logistic regressions were performed to determine the odds ratios (ORs) after including significant CAD risk factors: gender, age, smoking, and family history (FH) of CAD.

## 3. Results

A total of 695 subjects (22.3% females, mean age = 54 ± 11 years) with angiographically assessed stenosis were analyzed. The demographic and descriptive data of three stenosis groups are given in Table 1. Distributions of* APOE* genotypes and allele frequencies are presented in Table 2. Out of 695 analyzed subjects, we obtained final genotype calls for 672 samples. The distribution of* APOE* genotypes in 672 subjects was in the Hardy-Weinberg equilibrium (*P* = 0.721). Six samples with the 2/4 genotype were excluded (672 − 6 = 666) from the association analysis due to known opposite effects of the* E2* and* E4* on CAD. First, we assessed the association of* APOE *polymorphism among 666 subjects with percentage of CAD stenosis by evaluating the pattern of distribution of E2 (2/2 and 2/3), E4 (3/4 and 4/4), and E3 (3/3) genotype groups among three stenosis categories (<30%, 30–69%, and ≥70%) (Table 3). Specifically, the C-A trend test was used to detect whether the proportion of E4 or E2 increases linearly with the level of CAD severity when comparing with E3 group. The proportions of E4 (E3 versus E4) are 0.14, 0.22, and 0.24 for three stenosis categories (<30%, 30–69%, and ≥70%), which significantly increases linearly with the level of CAD severity (*P* = 0.01), while the proportion of E2 (E2 versus E3) does not increase linearly with the level of CAD extent. We further examined the effect of* APOE4* on the severity of CAD by performing logistic regression analysis. After adjusting for gender, age, smoking, and FH, the* APOE*∗*4* associated ORs were 1.61 (95% CI: 0.52–4.5; *P* = 0.377) and 2.16 (95% CI: 1.29–3.79; *P* = 0.005) in the 30–69% and ≥70% stenosis groups, respectively.

Plasma lipid levels (TC, HDL-c, and TG) data were available only in subset of subjects (*n* = 216) and so they were not considered as covariates in the main analysis. However, we performed a post hoc analysis on this subset of individuals with available lipid levels data. Only TG showed nominally significant difference between the three stenosis groups (*P* = 0.03), followed by a nonsignificant trend of difference observed for TC (*P* = 0.43) (Table 1). In the extended post hoc analysis model, we included TG and TC as covariates in addition to gender, age, smoking, and FH. The adjusted OR associated with the E4 genotype group to develop ≥70% stenosis was 1.61 (95% CI: 0.59–5.23; *P* = 0.38) in this subset of sample. For comparison, we also did post hoc analysis in this subset using only gender, age, smoking, and FH as covariates, as we did in the main analysis and found a similar outcome (OR = 1.64; 95% CI: 0.62–5.27; *P* = 0.35), suggesting that using lipids as covariates in the main analysis would have also given similar results in the total sample. We also examined the impact of three* APOE *genotype groups, E2 (2/2 and 2/3), E3 (3/3), and E4 (3/4 and 4/4), on plasma lipid levels in this subgroup of individuals using gender, age, smoking, and FH as covariates but found no significant association with TC (*P* = 0.16), HDL-c (*P* = 0.23), and TG (*P* = 0.18).

Next, we evaluated the association of the* APOE* polymorphism with the number of diseased vessels in the ≥70% stenosis group but found no significant association (Table 4).

## 4. Discussion

Coronary artery disease is a major public health problem in Pakistan and is considered as the most prevalent cause of death. One out of four middle-aged men and women equally has CAD [14]. Compared with developed countries, heart disease among South Asians is studied only at limited scale [15, 16]. The genetic basis of coronary heart disease (CHD) among Pakistanis is largely an unexplored area of research. In order to fill this research gap, we examined the association of* APOE* polymorphism with angiographically assessed coronary stenosis in Pakistan population.* APOE* is an established genetic risk factor for CAD in Europeans and other populations [17, 18]. We hypothesized that the common* APOE* polymorphism will also affect the CAD risk among Pakistanis. To the best of our knowledge, this is the first study that has examined the association of* APOE* polymorphism with angiographically assessed stenosis and the number of diseased vessels among Pakistanis. The frequency of the* APOE*∗*4 *allele in our sample was 11%, which is very similar to its reported frequency of 9% in a relatively older Pakistani control sample of 108 individuals [19] but slightly lower than the average frequency of 14% among Europeans [20].

Different epidemiologic studies have shown inconsistent results between* APOE *polymorphism and CAD severity and extent. We found significant association between* APOE*∗*4 *and CAD stenosis like most reported studies. The risk of having significant CAD measured by stenosis was two times higher among* APOE*∗*4* carriers than other genotypes. However, we did not observe an association between* APOE*∗*4 *and number of diseased vessels in our sample as a determinant of disease extent. In Women's Ischemic Syndrome Evaluation (WISE) study,* APOE*∗*4* was associated with both severity of stenosis and number of diseased vessels [3]. On the other hand,* APOE*∗*4* was found to be associated with number of diseased vessels and not with stenosis in a study conducted by Wang et al. [17]. The protective role of* APOE*∗*2* against CAD was not observed in our study, most likely due to its low frequency in this population. This is consistent with previous studies where the association of* APOE*∗*2* with CAD was only detected in large-scale datasets [5, 16]. Moreover, all the meta-analyses done so far contained a variety of covariates and the specific adjustment for different covariates was not clear [4, 10].

A limitation of our study is that cholesterol and other lipid values were available only in a subset of individuals, and in this subgroup* APOE* polymorphism did not show any association with lipid levels. However, we observed an expected trend of association of cholesterol levels among the three stenosis groups in this subgroup where highest levels of TC were observed in the ≥70% stenosis group, intermediate in the 30–69% group, and lowest in the <30% group (Table 1). In our post hoc analysis in this subgroup with lipid levels, the association of* APOE*∗*4* with CAD severity was independent of lipid levels, suggesting that we would have likely seen this independent association in the total sample, had the lipid data been available on all subjects.

In case of multifactorial disorders, it has always been challenging to establish a mechanistic relationship between a particular allele and disease development. Coronary artery disease results from a sequence of events starting from endothelial cell injury followed by sequestration of oxidized LDL particles, smooth muscle cell proliferation, and intense inflammatory reaction. In addition to the opposite effect of the* APOE*∗*4* and* APOE*∗*2* alleles on cholesterol levels, different* APOE* genotypes have diverse antioxidant properties too, among which* APOE*∗*4* is the least antioxidant [21]. Furthermore, the ancillary role of* APOE* in lymphocyte recruitment makes it an important candidate gene responsible for inflammation in CAD [22]. The* APOE* polymorphism with divergent properties at cellular level could predispose to CAD by using multiple pathways that needs further probing.

## 5. Conclusion

In conclusion, we report the first study with the aim of evaluating the genetic determinants of CAD in Pakistanis, which confirms that persons with* APOE*∗*4 *have significant risk of developing coronary artery disease in the Pakistani population.

## Figures and Tables

**Table 1:** Patient characteristics according to the percent of vessel's stenosis (*n* = 695).

Parameters	<30% (*n* = 157)	30–69% (*n* = 30)	≥70% (*n* = 508)	*P* value
Age (years)	52.47 (12.56)	55.63 (13.40)	54.42 (11.32)	0.14
BMI (kg/m^2^)	28.89 (4.82)	28.71 (3.09)	28.98 (4.65)	0.94
Gender (M/F)	120/37	26/6	394/112	0.82
Smoking (no/yes)	91/66	118/14	395/11	5.7*E* ^−07^
Family history (no/yes)	87/70	12/20	346/160	7.5*E* ^−05^
^*^TC (mg/dL)	188.58 (46.53)	198.19 (44.02)	200.01 (53.90)	0.43
^*^HDL-c (mg/dL)	40.53 (10.26)	34.21 (6.06)	39.18 (9.29)	0.1
^*^TG (mg/dL)	171.22 (61.54)	147.26 (38.95)	186.52 (62.12)	0.03

^∗^TC, HDL-c, and TG were measured on subset of 216 subjects.

TC: total cholesterol; HDL-c: high-density lipoprotein; TG: triglyceride.

**Table 2:** Distribution of the *APOE* polymorphism in the genotyped sample of 672 subjects.

Genotype/allele	Count	(%)
22	2	0.3
23	36	5.36
24	6	0.89
33	491	73.07
34	129	19.2
44	8	1.19
*APOE∗2 *	46	3.42
*APOE∗3 *	1147	85.34
*APOE∗4 *	151	11.24

**Table 3:** Distribution of the *APOE* polymorphism by stenosis groups.

	<30%		30–69%		≥70%	
	*N*	%	*N*	%	*N*	%
E2	9	6.16	2	6.90	27	5.50
E3	118	80.82	21	72.41	352	71.69
E4	19	13.01	6	20.69	112	22.81
Total	**146**		**29**		**491**	

Trend test (E2 versus E3)	*χ* ^2^ = 4*E* − 04; *P* = 0.98					

Trend test (E3 versus E4)	*χ* ^2^ = 6.378; *P* = 0.01					

**Table 4:** Distribution of the *APOE* polymorphism by the number of vessel diseases among subjects with ≥70% stenosis (*n* = 491).

	One vessel		Two vessels		Three vessels	
	*N*	%	*N*	%	*N*	%
E2	16	8.16	3	2.4	8	4.71
E3	133	67.86	94	75.2	125	73.53
E4	47	23.98	28	22.4	37	21.76
Total	**196**		**125**		**150**	

Trend test (E2 versus E3)	*χ* ^2^ = 2.52; *P* = 0.11					

Trend test (E3 versus E4)	*χ* ^2^ = 0.10; *P* = 0.74					
